# Medical and Physical Intelligence

**Published:** 1802-01

**Authors:** 


### MEDICAL AND PHYSICAL INTELLIGENCE.

### [FOREIGN AND DOMESTIC.]

*On the origin of amber.*—It is almoſt univerſally acknowledged that amber derives it origin from the vegetable kingdom, though it is not ſufficiently aſcertained, which ſubſtance does chiefly contribute to its formation, and every analogous fact which might throw light on this ſubject has, therefore, a particular intereſt for naturaliſts. Of this kind is the following experiment, made for that purpoſe by Profeſſor Hermbſtaed: "Pour into a china ſaucer, oil of amber or rectified petroleum, ſo much that it ſtands about one line high; and, having placed it on water, cover it with a glaſs bell filled with oxygen gas, and expoſe the whole for ſome months to the action of ſun-light. As the air in the bell is by degrees abſorbed, the water will riſe, and the oil become more weighty than before, and of a greater conſiſtency. On evaporating the reſt of the oil, by gentle heat, a reſinous ſubſtance remains, which proves extremely analogous to amber."—The following theory is grounded on this experiment: "The rock-oil is the production of interred animal, but particularly vegetable, ſubſtances; which, having come by means of its ſpecific lightneſs on the ſurface of water, abſorbs the oxygen of the atmoſphere and becomes inſpiſſated. As long as it remained viſcid, flies, gnats, ſpiders, and other inſects that happened to ſit down on it, and ſtraw and other particles floating in the air, having ſtuck to it, are, by degrees, incorporated with it as it grows harder. When the ſolidity of the inſpiſſated oil increaſes by the continued abſorption of oxygen it ſinks to the bottom in maſſes, which forms the ſtrata of amber.—The acid obtained from amber by dry diſtillation, ſeems not only to be admixed to it, but rather compoſed of a more intimate combination of a part of amber with oxygen."—The late Mr. Girtanner was of opinion, that amber might be an animal production, and he imagines it conſiſted of a kind of honey and wax that had been changed changed into amber by a fpecies of ants, *(formica rufa)* which, by the acid they contain, have oxydated and hardened the wax. This idea, however, though applicable in fome meafure to the fubterraneous amber, does not agree with that production in general, and his firft theory feems to be more preferable, as it has recourfe to the influence of the atmofphere, an agent fo univerfally extended and fo frequently employed by nature for its different operations. Nothing befides can be traced by dry diftillation which had any reference to its animal origin; and in cafe ants had contributed towards its generation, there fhould at leaft exift in it traces of azote, as in the acid of ants, &c. The experiment of Profeffor Hermbftaedt deferves, therefore, every attention, however infufficient it appears for explaining the origin of fubterraneous amber; but this fpecies is alfo lefs hard, and does not poffefs all the properties of water amber.

---

It is but too frequently the cafe, that for procuring credit to a favorite fyftem, and for making profelytes, men are biaffed towards meafures, difhonourable to themfelves and difadvantageous to the caufe which they endeavoured to promote. Such is the following cafe, which throws great blame on the violent and unreafonable Champions of the Brunonian Syftem. In a late number of Profeffor *Roshlaub's* Magazine for the Theory and Practice of Medicine, which betrays a vifible bias towards that fyftem, are inferted from an anonymous hand, feveral letters relating to the laft illnefs of the celebrated *Kotzebue*, in order to prove that this famous author had been faved from inevitable death by the exertions of a Brunonian Doctor. We are told, that the complaints of Mr. *Kotzebue* were confidered as hypochondriacal, and originating from an obftruction in the bowels, and that he had been treated by a great many renowned phyficians without experiencing any relief, till at laft, on the brink of death, he applied to a young doctor, who treating him according to the enlightened principles of the Brunonian Theory, fucceeded in curing Mr. *Kotzebue* in a proportionably fhort time, much to the fatisfaction of the Doctor and to the honour of that ftandard fyftem. Every particular of the cafe is fo accurately ftated, and even the prefcriptions added, that were afferted to have been ufed by his former phyficians, as to leave no room for doubting its authority. The indignation of the public muft therefore be the more raifed, when we are affured by Mr. *Kotzebue* himfelf, that the whole is nothing but a moft fhameful forgery, the anonymous author of which had purpofely made fuch a celebrated man the fubject of it, in order to draw the attention of the public on this cafe to a man fo univerfally efteemed for his dramatical compofitions. Mr. *Kotzebue*, however, declared himfelf firft in the Hamburgh newspapers againft the authenticity of that cafe, but he particularly fhewed afterwards, more fully, the matchlefs impudence of that forger of lies. After a ftatement of his illnefs, he affirms never to have employed any other phyfician than Dr. *Bluhm* of Riga, a very able practitioner, whofe plan of cure had been approved by the greateft phyficians of of Germany, *Hufeland*, *Zimmermann*, *Selle*, *Marhard*, &c. The chief ſymptoms of his malady were a febrile ſtate, a palpitation of the heart, and inſupportable anxiety of mind, emaciation of the body, want of ſleep and appetite, which he thought to have ariſen from ſome dietetical irregularities. By the ſkilful uſe of aperients, roborants, motion, travelling, &c. &c. Dr. Bluhm ſucceeded in reſtoring Mr. *Kotzebue* to health. The language which this gentleman uſes in his reply to the ſtatement inſerted in *Roshlaub's* Magazine, denotes the juſt indignation to which he was rouſed by the impudent aſſertions of the author, which he calls open lies. He deſires the editor of the above Magazine, for his honour, to unmaſk the impoſtor, as otherwiſe he might juſtly incur the ſuſpicion of being an accomplice in that impoſition. It is impoſſible to conceive how any perſon could take the courage of forging ſuch a caſe without fear of being diſcovered, had the author not hoped, from a rumour of the emperor Paul's diſpleaſure with *Kotzebue*, and his being exiled to Siberia, that this impoſture would remain buried in the ſnow and ice of that country.

---

A Tranſlatiou into Engliſh of Cuvier's Treatiſe on Comparative Anatomy is in great forwardneſs. It is executing at Paris, under Cuvier's directions, by an Engliſh phyſician qualified to do juſtice to the undertaking. A number of notes by Cuvier himſelf will be added to the two firſt volumes, which are not in the French edition. Theſe volumes will be ſpeedily publiſhed at Edinburgh; and the remaining volumes will appear as early as theſe, in the original.

---

A late Pupil of Profeſſor Blumenbach is now engaged in tranſlating his Compendium of Phyſiology, to which he means to add explanatory notes and illuſtrations; a labour which, from the utility of the work and the difficulty of procuring the original in England, he has been led to undertake.

---

The Gentlemen elected as Preſidents by the Phyſical Society, Guy's Hoſpital, for the enſuing ſeſſion, are, Dr. Haighton, Dr, Walſhman, Dr. Currie, Mr. Saumarez, Mr. A. Cooper, and Mr. Hardy.

The Gentlemen next in order to read Caſes, ſome of which are upon the table, are Meſſrs. Talbut, Suter, Crump, and Shaw.

H. Johnson *Secretary*.

---

On Tueſday, Jan. 19, 1802, at eight o'clock in the evening, Dr. Garnett will commence a Courſe of Lectures on Chemistry, comprehending all the modern diſcoveries in that ſcience, with its application to the different arts and manufactures, particularly pharmacy, medicine, and agriculture. The courſe will conſiſt of about forty Lectures, two of which will be delivered every week, viz. on Tueſday and Thurſday, at eight o'clock in the evening. A ſhort courſe of *Mineralogy* will be introduced in the day, which thoſe those who subscribe to the Chemical Course will be allowed to attend.

On Wednesday Jan. 20, at eight o'clock in the evening, he will commence a Course of Lectures on ZOONOMIA, or the Laws of Animal Life; in which will be given a popular view of the animal oeconomy, and the laws by which its different functions are regulated, with the methods of preventing and curing diseases. The object of the Lecturer will be to render this course interesting, not only to students of medicine, but to all who think the study of the human frame a subject worth their inquiry.

This course will consist of not less than fifteen lectures, one of which will be delivered on Wednesday evening at eight o'clock.

For particulars, application may be made to Dr. Garnett, No. 51, Great Marlborough Street.

---

Dr. BRADLEY's Course of Lectures on the Theory and Practice of Medicine, will commence on Tuesday, Jan. 12, 1802, at six in the afternoon, at the Lecture Room, No. 102, Leadenhall Street.

---

Dr. BATTY's Lectures on the Theory and Practice of Midwifery, will commence on Monday, January 11, 1802, at eleven o'clock in the forenoon, at his house in Great Marlborough Street.

---

Dr. DENNISON and Dr. SQUIRE, early in this month, will commence a Course of Lectures on the Theory and Practice of Midwifery, and the Diseases of Women and Children.

---

Dr. OSBORN's and Dr. CLARKE's Lectures on Midwifery, and the Diseases of Women and Children, will be given as usual, at Dr. Clarke's house in New Burlington Street. The first of the two Spring Courses will begin on Monday, January 24, 1802, at half past ten in the morning; and the Lectures will be continued every day, at the same hour, for the convenience of Students attending the Hospitals.

---

Mr. BLAIR will commence a Course of Clinical Lectures on the Diseases and Operations of Surgery, on Tuesday the 26th of January, at eight o'clock in the evening, at the Bloomsbury Dispensary, in Great Russel Street.

---

Mr. CHEVALIER, Surgeon to the Westminster General Dispensary, will begin his Spring Course of Lectures on the Principles and Operations of Surgery, on Monday, Feb. 1, at seven o'clock in the evening, at the Dispensary in Gerard Street, Soho; where printed particulars may be had, or at his house in South Audley Street, Grosvenor Square.

---

Mr. NORTHCOTE, formerly from Plymouth, is now painting a Portrait of Dr. Jenner, at the request and expence of the Medical Society of Plymouth, and the Picture is immediately to be engraved by an eminent Artist.

